# Diffractive and Interferometric Characterization of Nanostructured Photopolymer for Sharp Diffractive Optical Elements Recording

**DOI:** 10.3390/polym10050518

**Published:** 2018-05-10

**Authors:** Roberto Fernández, Sergi Gallego, Yasuo Tomita, Inmaculada Pascual, Augusto Beléndez

**Affiliations:** 1 Instituto Universitario de Física Aplicada a las Ciencias y las Tecnologías, Universidad de Alicante, P.O. Box 99, E-03080 Alicante, Spain; sergi.gallego@ua.es (S.G.); pascual@ua.es (I.P.); a.belendez@ua.es (A.B.); 2 Departamento de Física, Ingeniería de Sistemas y Teoría de la Señal, Universidad de Alicante, P.O. Box 99, E-03080 Alicante, Spain; 3 Department of Engineering Science, University of Electro-Communications, 1-5-1 Chofugaoka, Chofu, Tokyo 182-8585, Japan; ytomita@ee.uec.ac.jp; 4 Departamento de Óptica, Farmacología y Anatomía, Universidad de Alicante, P.O. Box 99, E-03080 Alicante, Spain

**Keywords:** photopolymers, optical recording material, diffractive optical element, nanoparticle-polymer composite, nanoparticles

## Abstract

We study the behavior of a nanoparticle-polymer composite (NPC) material, based on a thiol-ene monomer system, working with long grating spacing. Thus, we evaluate the suitability of the NPC for storing complex diffractive optical elements with sharp profiles, such as blazed gratings. Using holographic methods, we measure the “apparent” diffusion of the material and the influence of the spatial period on this diffusion. The applicability of this material in complex diffractive optical elements (DOEs) recording is analyzed using an interferometric method. Supported by the results of this analysis, we record blazed gratings with different grating spacing and measure the maximum diffraction efficiency (DE) achieved. The results show that NPC has a good behavior in this range of spatial frequencies.

## 1. Introduction

Photopolymerizable nanoparticle-polymer composites (NPCs) are a holographic dry composition in which inorganic nanoparticles, such as TiO_2_, SiO_2_, ZrO_2,_ nanozeolites [1,2,3,4,5], and organic nanoparticles [6], are uniformly dispersed in organic host monomers that are capable of chain-growth polymerization. During the polymerization process, a light-induced redistribution of the monomer and nanoparticles takes place in the bright and dark regions of the light pattern, respectively. This is due to the mutual diffusion process of the monomer and nanoparticles driven by the polymerization process [1,7,8].

The inclusion of inorganic nanoparticles, which have a higher refractive index than that of the formed polymer, leads to important improvements in refractive index modulation. Other improvements include the suppression of polymerization shrinkage and a high thermal stability [1,2,9]. Such improvements may be combined by the incorporation of liquid crystal molecules into an NPC material for electro-optic control [10,11,12] of the formed holographic grating recorded in the NPC material.

In previous work [13], we explored the electro-optic capabilities of a holographic polymer dispersed liquid crystal (HPDLC) in working with low spatial frequencies.

We found that it was difficult to increase the refractive modulation amplitude due to its large monomer diffusivity, and also to reduce polymerization shrinkage during recording for the realization of low spatial frequency diffractive optical elements (DOEs) with sharp profiles. For this reason, we investigate the electro-optic characteristics of DOEs recorded in the NPC material incorporated with liquid crystals in the low spatial frequency regime. Along this line, our present work examines the holographic recording properties of NPC gratings possessing large grating spacing (low spatial frequencies).

For holographic recording, the high performances of NPCs based on thiol-ene/thiol-yne photopolymerization have been demonstrated and the recording dynamics have been analyzed in detail [14,15,16,17]. Further, new chemistry systems and formulations for thiol-ene systems have been developed. Recently, Bowman et al. proposed thiol-X click chemistry, with simple processability and environmental friendliness [18], and a 1,3-bis(phenylthio)-2-propyl acrylate (BPTPA) monomer, a writing monomer capable of reaching a refractive index modulation up to 0.029 [19]. In this work, we carry out a novel analysis of the recording dynamics of thiol-ene-based NPC gratings at low spatial frequencies, for which we follow the same procedure as those previously used for other families of photopolymers, such as polyvinyl alcohol acrylamide (PVA/AA) based photopolymers, one of the most widely-studied photopolymers due to its good characteristics [20,21,22], Biophotopol [22,23], or HPDLC [13,22]. PVA/AA and Biophotopol materials have also demonstrated good results working with complex DOEs, obtaining good results from the material and high diffraction efficiencies (DEs). These values were near to their maximum, if we consider the losses introduced by the setup, which will be commented on below. Therefore, it is interesting to use PVA/AA, Biophotopol and HPDLC as a reference to compare to the NPC material for these applications. The results of this study show the suitability of the NPC material to work, not only in a holographic regime, but also to store many complex DOEs, such as blazed gratings [24] or diffractive lenses [25]. We start with a post-exposure evolution analysis to measure the monomer’s “dark” diffusion after a short recording exposure. In the post-exposure process, there is no polymerization and only the monomer’s diffusion is responsible for changes in the profile. Post-exposure evolution analysis is also performed to investigate whether or not monomer dark diffusion depends on grating spacing, extending the analysis to different spatial period gratings. Moreover, using the zero-frequency analysis method [26], we can complete the material’s characterization. In this zero-frequency limit, we avoid the monomer’s diffusion effects and directly measure different parameters, such as the polymerization rate (*F*_R_) [27,28]. Through the zero-frequency analysis method, we study the capabilities of the NPC material to reach a phase depth of 2π. This is an important point because this phase depth is required for complex DOEs. Furthermore, we perform a recording of a complex element, a blazed grating, in the NPC material. This is done by using a liquid crystal on a silicon (LCoS) microdisplay based spatial light modulator (model PLUTO, Holoeye, Berlin, Germany), which is turned into a phase element once projected on the NPC material.

## 2. Experimental Setup

**Figure 1:**
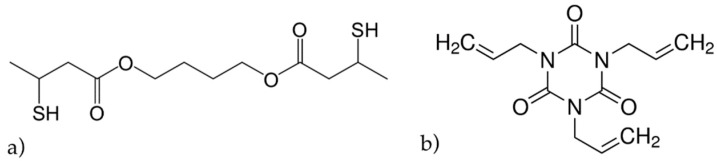
Chemical structures of dithiol (**a**) and triallyl (**b**) monomers.

**Figure 2:**
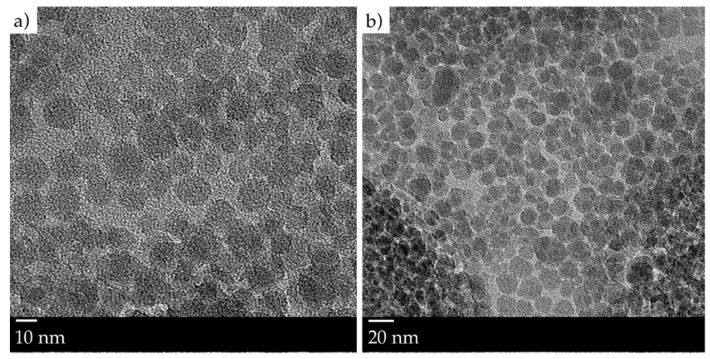
TEM images of SiO_2_ nanoparticles in MIBK at 10 nm (**a**) and at 20 nm (**b**).

Our NPC monomer system was composed of a stoichiometric mixture of thiol-ene monomer, containing an allyl-triazine-ene monomer, triallyl-1,3,5-triazine-2,4,6(1H,3H,5H)-trione (Aldrich, St. Louis, MI, USA), and a dithiol monomer, 1,4-bis(3-mercaptobutyryloxy)butane (Showa Denko K.K., Tokyo, Japan), of which the chemical structures are shown in Figure 1a,b. Ten- to fifteen-nanometer SiO_2_ nanoparticles, dispersed in methyl isobutyl ketone (MIBK, Nissan Chemical Industries Ltd., Tokyo, Japan) at a concentration of 30 wt % were dispersed to the thiol-ene monomer blend. Transmission electron microscopy (TEM) images of the nanoparticles, at 10 and 20 nm, are shown in Figure 2a,b respectively. The refractive index difference between SiO_2_ nanoparticles and the formed thiol-ene polymer was close to 0.1, which helps to obtain large values of the refractive index modulation. The use of nanoparticles also improves the thermal stability of the gratings recorded in the NPC material, as mentioned earlier [9]. The efficient polymerization of the NPC material using a green laser was achieved by the addition of 2 wt % of titanocene organo-metallic complex (Chivacure 534, Chitec, Taibei, Taiwan) in combination with 2.5 wt % benzoyl peroxide (BzO_2_, Aldrich, St. Louis, MI, USA) [14,15,16].

**Figure 3:**
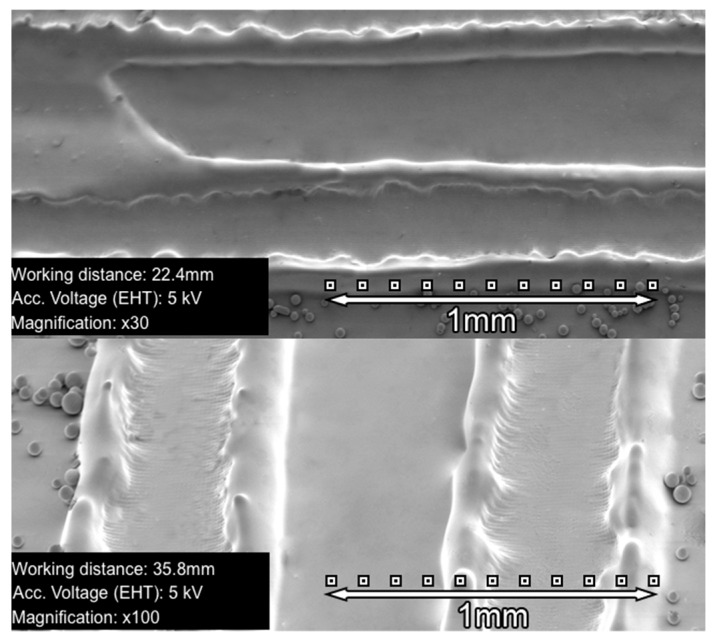
SEM images of a 672 µm blazed grating recorded in a 30-µm thickness NPC material.

The mixed syrup was dropped onto a glass substrate and dried in an oven at 55 °C for 20 min to eliminate the MIBK solvent. Then, another glass substrate was placed to cover the syrup. We used 20–30 µm glass microspheres (White Scientific) as spacers between the two glass substrates. Figure 3 shows SEM images of a blazed grating of 672 µm, recorded in 30-µm thickness NPC material. These images were taken from uncovered material. We made the material used between the glass substrate and the cover slip and removed the glass substrate after recording. In the removal process, the grating was slightly stretched, as seen in Figure 3. In this figure, the glass microspheres, used as spacers, can be observed at the sides of the grating.

**Figure 4:**
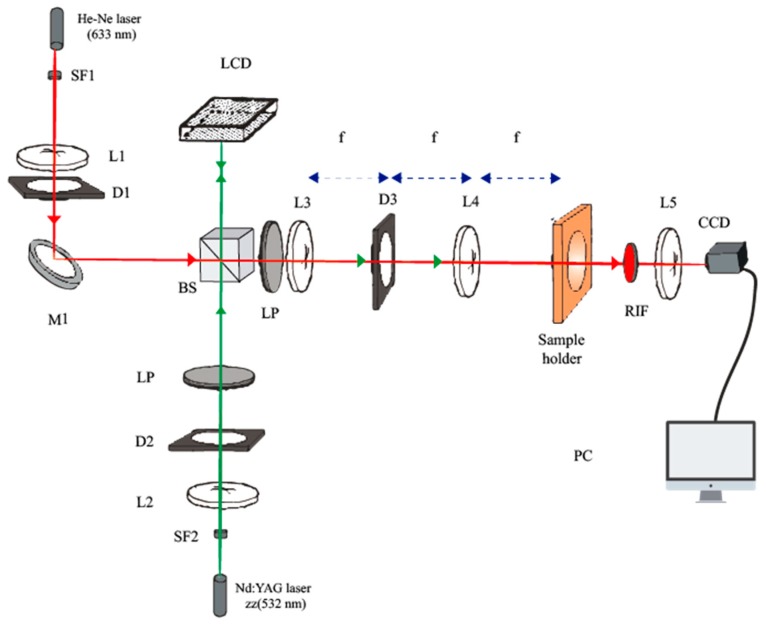
Experimental setup. BS: beam splitter, Mi: mirror, SFi: spatial filter, LP: lineal polarizer, Li: lens, Di: diaphragm, RIF: red interference filter and PC: data recorder.

To record and evaluate the DOEs in the NPC material, we used the setup shown in Figure 4, which consisted of two beams. The recording green beam was formed using a solid-state laser (model Verdi Nd:YVO_4_, Coherent, CA, USA), operating at a wavelength of 532 nm (green light). The DOE pattern was introduced by the LCoS spatial light modulator (SLM), which was located on the recording arm. Amplitude modulation was produced using the two polarizers (LPs) placed on both sides of the device. Then, to image the intensity distribution generated by the SLM onto the recording material, we used a 4f system, which gives direct access to the Fourier plane. Two identical lenses (L3, L4) had a common focal point, where a diaphragm (D3) was placed. Thus, the distance between input plane and image plane was 4 times the length of f [29]. We measured DEs in real time, defined as:

(1) $$\mathrm{DE}=\frac{I_{i}}{I_{I}}$$

where *I*_i_ is the intensity of the diffraction orders and *I*_I_ is the incident intensity.

The system was made up of two beams. An s-polarized He-Ne laser operating at a wavelength of 633 nm, where the NPC material had no light absorption, was used to analyze changes due to the photopolymerization in real-time. This beam was collimated before it reached the NPC material and its aperture was controlled using a diaphragm (D1). Both beams passed through a non-polarizing beam splitter (BS), following the same path. Behind the NPC material, there was a red interference filter (RIF) so that only the information from the red beam, the analyzing beam, was captured on a high dynamic range CCD camera (model pco.1600, pco.imaging, Kelheim, Germany), which was located at the end of the setup, at a resolution of 1600 × 1200 and a pixel size of 7.4 µm × 7.4 µm. In front of the CCD camera a lens (L5) was used to separate the diffraction behind the material and obtain the Fraunhofer diffraction pattern. The intensity pattern to be recorded into the NPC material was also evaluated, using the CCD camera to record the pattern imaged by the SLM plane. The recording intensity used was 0.2 mW/cm^2^.

## 3. Results and Discussion

In this section we present various experimental results for the DE achieved with the NPC material. The different experiments carried out were described in the Introduction. As DEs of different diffracted orders in the Fraunhofer domain are given by the Bessel functions [30], a comparison of the theoretical DEs with those measured experimentally gives the linearity in the response of the NPC material. The results are discussed in comparison to those obtained for PVA/AA, Biophotopol and HPDLC materials.

### 3.1. Post-Exposure Evolution

In this section, we will discuss the post-exposure evolution of the NPC material. Following the method proposed in Reference [27], we can fit the value of the apparent monomer diffusion. Due to the confinement of the NPC material between two glass substrates, we cannot measure separately the changes in the surface and the internal changes through an index matching system, as we did previously for PVA/AA and Biophotopol based materials.

**Figure 5:**
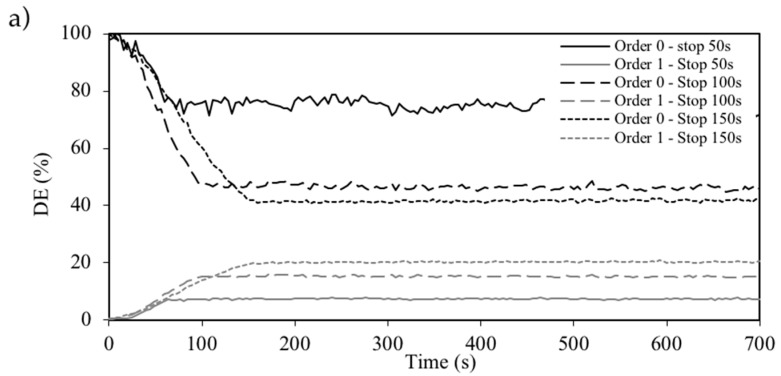

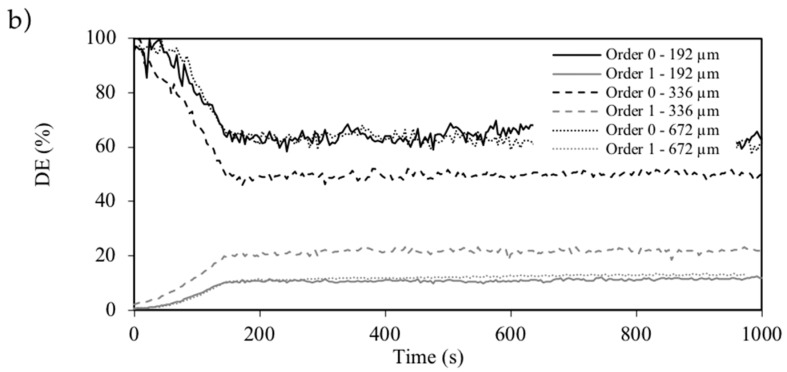
(**a**) DE of zero and first orders as a function of time for an exposure of a 720 µm sinusoidal grating over 50, 100 and 150 s and the post-evolution. (**b**) DE of zero and first orders as a function of time for an exposure of 192, 336 and 672 µm sinusoidal gratings during 150 s and the post-evolution.

**Figure 6:**
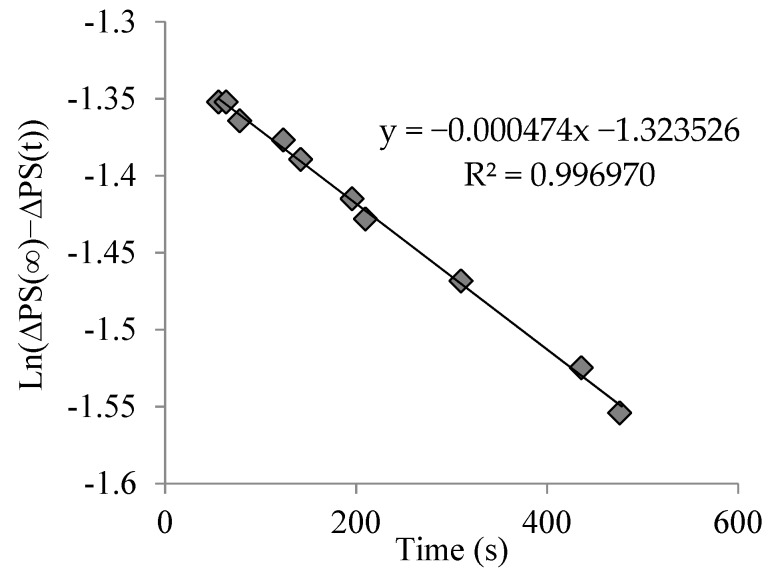
Fitting to obtain diffusivity value for an exposure time value of 150 s.

We exposed a 30-µm in thickness layer to a 168-µm sinusoidal grating during different exposure times of 50, 100 and 150 s, then switched off the recording green laser and studied the evolution of the NPC material. Then, following the method applied in Reference [28], we calculated the value of diffusivity through fitting using the following relation:

(2) $$\ln(\mathrm{PS}(x,\;t\to \infty )-\mathrm{PS}(x,\;t))=\ln(\mathrm{PS}(x,\;t\to \infty ))-\frac{D\cdot 4\pi^{2}\cdot t}{\Lambda^{2}}$$

where PS is the phase shift, PS(x, t→∞) is the phase shift for long times when the grating becomes stable, *D* is “apparent” diffusion, and Λ represents the grating period. In this case, after recording, the DE of the zero and first order remained practically constant. Figure 5a shows the post-exposure evolution of the material after 50, 100, 150, 350, and 400 s of exposure. The fitting for the grating, stopped after 150 s of exposure time to obtain the diffusivity value, and is shown in Figure 6. The diffusivity values measured were (2 ± 1) × 10^−9^ cm^2^/s, which are higher than those measured for the PVA/samples with index matching system [21], but much lower when compared with samples without the index matching system, in which values around 1^−8^ cm^2^/s were obtained [21,31]. As the experiments were conducted several times, the error estimated (±1 × 10^−9^ cm^2^/s) was due to the repeatability and the existing ambiguity when determining the PS(x, t→∞).

We also studied the influence of grating spacing on apparent diffusion, because an increase of grating spacing may increase the apparent diffusion rate [16]. This is not desirable when working with DOEs composed by different spatial periods, such as diffractive lenses [25]. Figure 5b shows the post exposure evolution of 192, 336, and 672 µm sinusoidal gratings, recorded over 150 s and their post-exposure evolutions. In this case, the diffusivity values measured were near those previously measured: (2 ± 1) × 10^−9^ cm^2^/s at grating spacings of 192 and 336 µm, and (8 ± 1) × 10^−9^ cm^2^/s at a grating spacing of 672 µm. Therefore, we found that there was no strong influence of grating spacing on the apparent diffusion.

### 3.2. Phase Shift and Diffraction Efficency

The analysis of the recording of blazed gratings in PVA/AA, Biophotopol and HPDLC materials [13,24] was carried out in previous research. Blazed grating is a complex DOE, which presents a sharp profile with abrupt changes. This profile has many applications in communications and a theoretical maximum DE near 100%.

Firstly, to see the capabilities of the material to reach a phase depth of 2π, we used zero spatial frequency analysis [20,32]. We illuminated an area (1 cm^2^) and, using an interferometric method, we measured the phase shift (PS) between the exposed and non-exposed zones as a function of time without the influence of diffusion. In References [33,34], *F*_R_ has been studied and simulated for thiol-ene systems. In our case, the polymerization rate depended on the photon dose, the recording intensity, and the fraction of light absorbed by the photoinitiator. Following the procedure developed in Reference [20], we obtained *F*_R_ as a function of the experimental values of PS during recording and the phase of saturation (PS_∞_):

(3) $$\ln\left(1-\frac{\mathrm{PS}\left(t\right)}{{\mathrm{PS}}_{\infty }}\right)=-F_{R}\text{·}t$$

**Figure 7:**
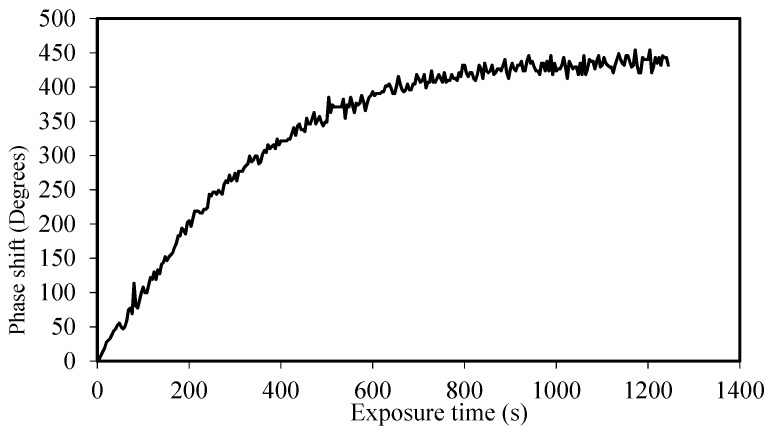
Phase shift as a function of exposure time for a 30-µm thickness material.

**Figure 8:**
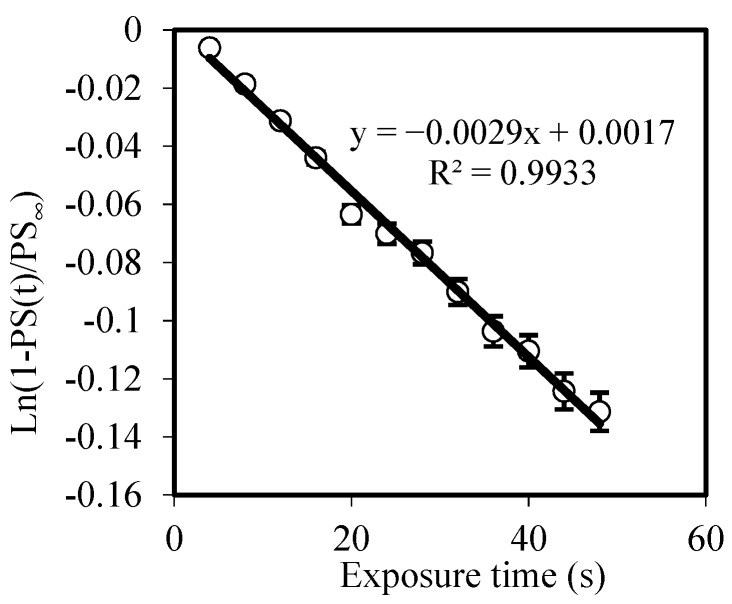
Fitting derived from Equation (3) to extract the value of polymerization rate (*F*_R_).

Figure 7 shows the phase shift as a function of exposure time for a 30-µm thickness sample using an intensity of 0.2 mW/cm^2^. As seen in Figure 7, the phase shift between the exposed and non-exposed zones was near 450° over the 2π needed for complex DOEs recording. The maximum phase shift was reached after 1000 s of exposure time; this long exposure means that the polymerization time of the NPC material was significantly slower than the reaction time of other analyzed materials, such as AA/PVA, Biophotopol, or HPDLC, where all the monomer is consumed after 100 or 200 s with the same intensity. This can be due to the significantly lower absorbance at the recording wavelength of the NPC material [15] compared to the recording wavelength of the other families of materials analyzed [23,35], causing this slower reaction time. Figure 8 shows the fitting of Equation (3) to obtain the value of F_R_. The value obtained for this polymerization rate was 2.9 × 10^−3^ s^−1^. It is also worth mentioning the high correlation value of the fitting, which indicates a good agreement between theory and experimental results.

**Figure 9:**
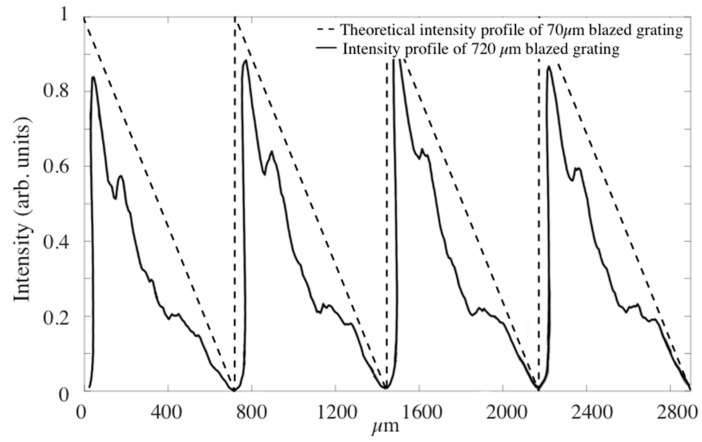
Intensity profile provided by the LCoS across a vertical line of the image recorded using the CCD camera on the material plane and theoretical profile of blazed grating.

Since the material exhibited the capability of reaching a 2π phase shift for 30-µm thickness samples, we proceeded to analyze the recording of blazed gratings. First, we used the CCD camera to evaluate the intensity pattern imaged from the plane of the modulator. Figure 9 shows the intensity distribution provided by LCoS across a vertical line, compared to the theoretical one. The experimental intensity distribution was extracted from a vertical line of the image of a 720 µm period grating, recorded by the CCD camera. It is noticeable that the experimental intensity distribution presents a smoothing of abrupt edges, due to the low pass filtering introduced by the setup.

**Figure 10:**
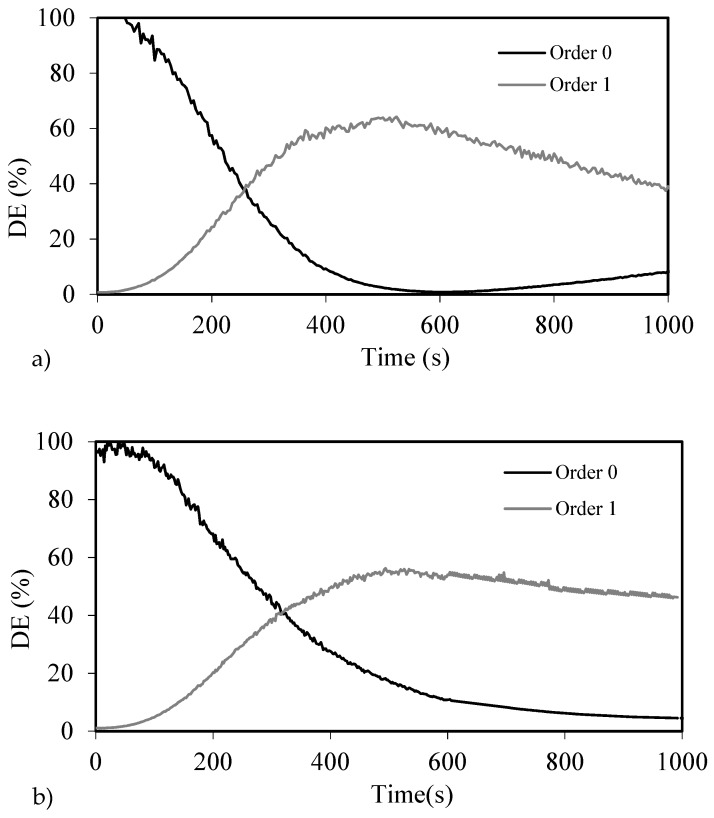

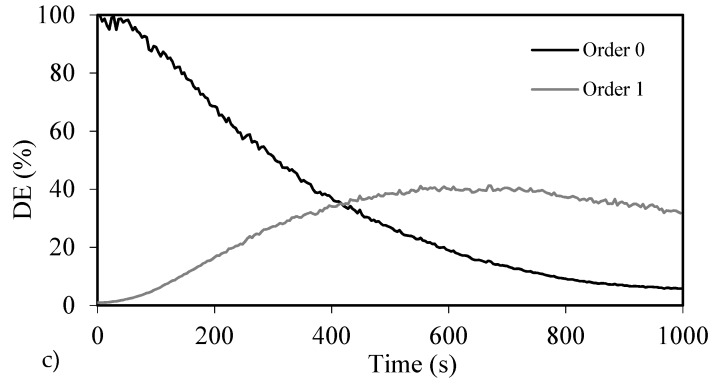
Diffraction efficiency of zero and first orders as a function of time for 720 µm (**a**), 336 µm (**b**) and 168 µm (**c**) blazed gratings recorded onto NPC material.

We recorded gratings of different spatial periods in 30-µm samples and checked the DE as a function of exposure time. Figure 10a–c shows DE as a function of time for 720, 336, and 168 µm gratings, respectively, using a recording intensity of 0.2 mW/cm^2^. The maximum value of DE was obtained for the 720-µm blazed grating, which reached close to 65% of DE after 500 s of exposure. This value is near the maximum achievable by the material, accounting for the low pass filtering which reduces the maximum DE obtainable by more than 20% [24]. In this case, the reaction time shown by the NPC material was slower compared to the other materials studied (PVA/AA based material, Biophotopol, HPDLC).

The effects of the diffusion are stronger for shorter grating spacings, as well as the effect of the low pass filtering commented above. These effects are noticeable in the maximum DE obtained, which was lower for smaller spatial periods, near 55% for the 336 µm grating and 40% for the 168 µm grating. This diffusion was also affected by the viscosity of the material, which depends on the drying conditions and modifies the diffusion coefficient, as demonstrated previously for different photopolymers and grating periods [36]. However, these DEs results are good, considering that due to the low pass filtering it is not possible to reach the 100% of theoretical DE.

## 4. Conclusions

We analyzed the behavior of an NPC material working with long grating spacing (low spatial frequencies) and its suitability to store complex DOEs, such as blazed gratings. We have found that the value measured for the diffusion of the material, (2 ± 1) × 10^−9^ cm^2^/s, is significantly lower than those measured for materials without using the index matching system. We have also verified that the grating spacing does not have a strong influence on the diffusion, because we have obtained similar values of diffusivity for three different grating spacings.

This study was completed with a zero-frequency limit analysis, without diffusion effects, which showed the capability of the NPC material to reach a phase depth higher than 2π. This analysis also permitted us to fit the value of the polymerization rate, 2.9 × 10^−3^ s^−1^.

Supported by the phase depth value obtained, we recorded blazed gratings of various grating spacing. We obtained a maximum DE of 65% for the 720 µm grating, near the maximum achievable given the low pass filtering introduced by the setup. This first approach has shown the good behavior of the NPC material and set it up as a good candidate for this range of spatial frequencies, keeping open the possibility of continued research with the NPC material in this range.
